# High-Throughput Particle Manipulation Based on Hydrodynamic Effects in Microchannels

**DOI:** 10.3390/mi8030073

**Published:** 2017-03-01

**Authors:** Chao Liu, Guoqing Hu

**Affiliations:** 1 CAS Key Laboratory for Biological Effects of Nanomaterials and Nanosafety, CAS Center for Excellence in Nanoscience, National Center for NanoScience and Technology, Beijing 100190, China; liuchao@imech.ac.cn; 2 State Key Laboratory of Nonlinear Mechanics, Beijing Key Laboratory of Engineered Construction and Mechanobiology, Institute of Mechanics, Chinese Academy of Sciences, Beijing 100190, China; 3 School of Engineering Science, University of Chinese Academy of Sciences, Beijing 100049, China

**Keywords:** particle manipulation, inertial lift, viscoelastic effects, microfluidics, lab on a chip, high throughput

## Abstract

Microfluidic techniques are effective tools for precise manipulation of particles and cells, whose enrichment and separation is crucial for a wide range of applications in biology, medicine, and chemistry. Recently, lateral particle migration induced by the intrinsic hydrodynamic effects in microchannels, such as inertia and elasticity, has shown its promise for high-throughput and label-free particle manipulation. The particle migration can be engineered to realize the controllable focusing and separation of particles based on a difference in size. The widespread use of inertial and viscoelastic microfluidics depends on the understanding of hydrodynamic effects on particle motion. This review will summarize the progress in the fundamental mechanisms and key applications of inertial and viscoelastic particle manipulation.

## 1. Introduction

**Figure 1:**
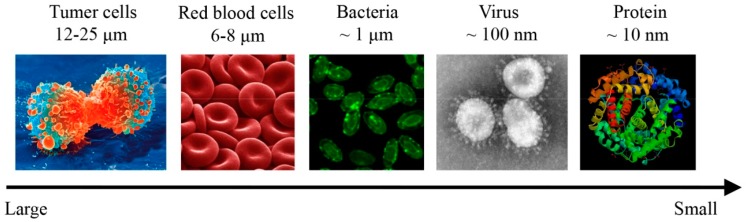
The size ranges of typical types of bioparticles. Cells, bacteria, virus, and biomacromolecules are particles with sizes ranging from tens of micrometers to tens of nanometers.

Cells, bacteria, virus, and biomacromolecules are particles with sizes ranging from tens of micrometers to tens of nanometers (Figure 1). The precise manipulation of these bioparticles is essential to various research and application fields [1,2]. For example, the detection of circulating tumor cells (CTCs) is essential to the early prognostic of cancer and the research on determination of their phenotype and genotype, which can provide better understanding of metastasis process and better guidance of cancer therapy [3,4,5]. However, CTC detection is challenging due to extremely low CTC concentration (1–100 CTCs/mL of blood) and large blood cell background. Therefore, a prior separation step of CTCs with high-throughput is critical for accurate and sensitive detection.

Conventional separation techniques often rely on immunocapture. Although useful for clinical and research purposes, cells may suffer irreversible damage during labeling. Moreover, the purity and retrieval of CTCs could be affected by the significant variation of the presence of specific biomarkers such as epithelial cell adhesion molecule (EpCAM) or human epidermal growth factor receptor 2 (HER-2) on CTC surface, even for the same tumor type [6,7]. For example, CellSearch^®^ system uses magnetic nanoparticles coated with anti-EpCAM antibodies to capture CTCs from human blood. Although being the only Food and Drug Administration (FDA)-cleared tested technique for capturing and enumerating CTCs, CellSearch^®^ system was recently discontinued due to its high miss rate and low CTC viability. The system fails to identify CTC from 7.5 mL blood samples (the manufacturer’s protocol) for nearly half of the 430 tested cancer patients. To achieve a reliable detection, at least 30 mL peripheral blood instead of 7.5 mL has to be collected [8], requiring the ability of high-throughput sample handling [5].

In addition to the immunocapture-based techniques, researchers have tried to enrich and isolate cells and particles based on their physical properties, such as size, shape, deformability, density, compressibility, charge, polarizability, and magnetic susceptibility. Exploiting these biomarkers, extensive methods have been developed, including hydrodynamics-based methods [9], acoustophoresis [10], dielectrophoresis [11], magnetophoresis [12], optophoresis [13], and centrifugation [14] (Table 1). Among them, the manipulation techniques using hydrodynamic effects in microchannels have attracted increasing attention because of the merits from their simple implementation and often high throughput. Specifically, in this review, we will focus on discussing the recent innovations for the hydrodynamic manipulation of particles. To do so, we will first briefly describe the techniques based on particle’s following streamlines at low Reynolds number *Re* (Re=ρUD/η, where U is the characteristic velocity, *D* the characteristic channel dimension, ρ the fluid density, and η the fluid dynamic viscosity). Their advantages and limitations will serve as an introduction and motivation for the more recent techniques based on particle’s cross-stream migration caused by hydrodynamic lift forces. The inertial microfluidics is discussed in detail, with emphasis on the fundamental mechanisms and the applications in high-throughput particle manipulation. Examples of specific microfluidic devices will be described and organized regarding their functions and designs, followed by the latest progress in the fundamentals of particle migration that provide better guidelines for the inertial microfluidic communities. We further discuss the more recent innovations in particle manipulation using viscoelastic effects, which enable more flexible working flow conditions and applicable particle sizes.

## 2. Particle Manipulation Based on Low-Reynolds Number Hydrodynamic Effects

**Figure 2:**
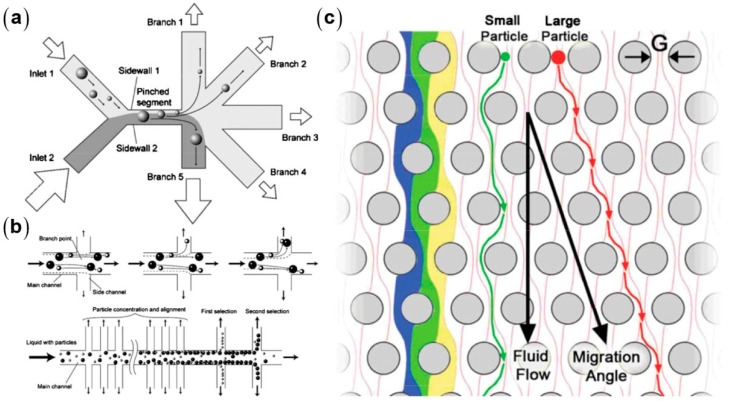
Hydrodynamic particle separation: (**a**) Pinched flow fractionation [15] (Reproduced with permissions from Takagi et al., Lab on a Chip; published by Royal Society of Chemistry, 2005); (**b**) hydrodynamic filtration [17] (Reproduced with permissions from Yamada et al., Lab on a Chip; published by Royal Society of Chemistry, 2005); and (**c**) deterministic lateral displacement [29] (Reproduced with permissions from Inglis et al., Applied Physics Letters; published by American Institute of Physics Publishing, 2004).

These techniques include pinched flow fractionation (PFF) [15,16], hydrodynamic filtration [17,18], deterministic lateral displacement (DLD) [19,20], and hydrophoresis [21,22,23,24], which presume that the particle center will strictly follow the streamline at low *Re* (Figure 2). They are typically based on the fluid and particles interacting with microstructures to separate particles by size, shape, and deformability [25,26]. In PFF, the suspension of particles with different sizes is introduced from a side microchannel into the main microchannel. The flow rates are tuned to pinch particles into a narrow stream and adjacent to the wall, making the centers of all particles located at different streamlines according to their size difference. In a sudden expansion at the downstream, the particles with different sizes are separated with a large lateral distance relative to expansion. With a similar operating principle, hydrodynamic filtration introduces particle suspension from a single inlet of a microchannel with multiple perpendicular branched outlets. The initially dispersed particles are gradually aligned along the side walls by repeatedly withdrawing a small portion of fluid from the main stream through the branched outlets. In the downstream of the microchannel, smaller particles enter into the branched outlets earlier than larger ones because the smaller particles locate closer to the side walls. Therefore, this technique enables particle separation and concentration simultaneously. To determine the size cutoff of the filtered particles, this technique requires precise microchannel fabrication to finely control the velocity profile and flow rate ratio at the branch point. Different from the above two techniques, DLD relies on a micropillar array in which each pillar row is laterally offset with respect to the predecessor row with a finely tuned distance. This design creates different streamline groups that move in the mainstream direction or along the offset sides of the predecessor pillars, depending on the pillar-streamline distance. Due to the steric hindrance of the pillar wall and the streamline following of microparticles, particles smaller than a critical size repeatedly move through the pillar gaps in an average mainstream direction, whereas particles larger than the critical size are laterally “bumped” along the offset direction due to the larger particle-pillar distance. The lateral distance between the large and small particles accumulates after multiple pillar rows, resulting in a final separation. Using these techniques, label-free separation has been achieved for diverse blood cell types [15,18,27] and CTCs [28]. It is worth mentioning that DLD has a very good size resolution, but with a very low throughput. Very recently, size sorting of nanoparticles down to 20 nm and exosomes has been demonstrated using DLD at the flow rates of ~10 nL/h [20]. Hydrophoresis is a separation technique using the particle motion influenced by a microstructure-induced pressure field. It typically uses microfluidic devices containing slanting obstacles to generate a lateral pressure gradient that induces helical recirculation and consequently focuses microparticles at different lateral positions depending on their size or deformability. Hydrophoresis also has a high separation resolution, enabling the discrimination of microparticles with diameter differences as small as 7.3% [21]. PFF and DLD often require sheath flows, which help to obtain high separation resolutions. However, sheath flows might cause several challenges: (1) the branched inlets for sheath flow make poor device parallelizability; (2) the control and operation become complex with sheath flows; and (3) the sample throughputs are limited due to the large sheath–sample flow ratio. Therefore, these techniques are commonly used for handling small volumes of samples.

## 3. Inertial Manipulation of Particles

**Figure 3:**
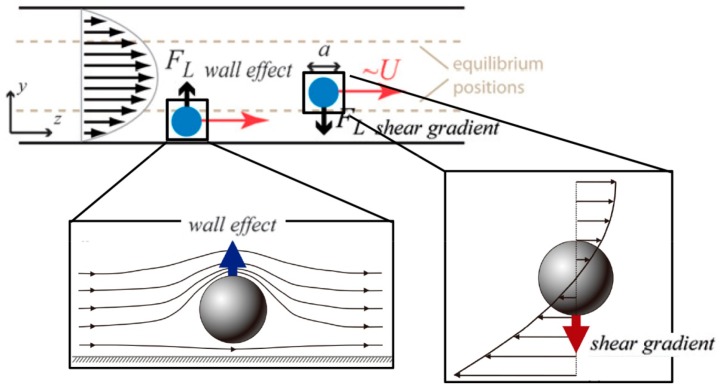
General mechanism of inertial lift: shear-gradient-induced lift arising from the curvature of Poiseuille velocity profile and wall-induced lift arising from the wall repulsion [32,33]. (Reproduced with permissions from Martal et al., Annual Review of Biomedical Engineering; published by Annual Reviews, 2014 and Reproduced with permissions from Amini et al., Lab on a Chip; published by Royal Society of Chemistry, 2014).

Different from the aforementioned methods, inertial microfluidics works by driving particles cross-stream migration utilizing inertial lift arising from the fluid flow nonlinearity at finite *Re* [30]. Due to its enhanced strength with increasing flow rate, inertial lift is favorable for high-throughput particle manipulation. Suspended in a pressure-driven flow of finite *Re*, particles will laterally migrate due to the acting inertial lift, which can be briefly attributed to the competition between two effects: (1) the shear-gradient-induced lift arising from the curvature of the Poiseuille velocity profile that drives the particle toward the wall; and (2) the wall-induced lift that pushes the particle away from the wall (Figure 3). The magnitude of inertial lift scales as $F_{L}\propto \rho U^{2}a^{4}$ [30,31]. Although fluid inertia was traditionally thought to be insignificant in microfluidic systems, microchannels are more favorable to realize deterministic particle control than macroscale channels. To produce a sufficient large shear gradient, very high flow speeds are required in macroscale channels, resulting in turbulent flows where the precise control is broken down. By contrast, microchannels can still generate large shear gradients even at relatively low flow speeds.

### 3.1. Inertial Particle Focusing in Straight Microchannels

**Figure 4:**
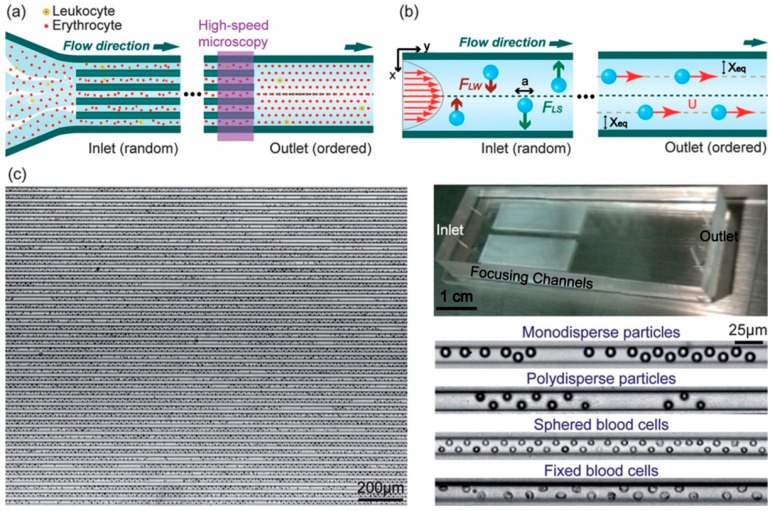
Inertial particle ordering in straight microchannel: (**a**) schematics shows a high-throughput cell ordering in parallel straight microchannels; (**b**) the competition of two opposite lift forces results in particle focusing at specific lateral positions; and (**c**) the massively parallel inertial microfluidic device and zoom-in images of particles and blood cells flowing in a single microchannel [39] (Reproduced with permissions from Hur et al., Lab on a Chip; published by Royal Society of Chemistry, 2010).

The focusing of cells is essential to their detection and characterization [34,35,36], whose accuracy and sensitivity highly depend on the focusing quality (the percentage of particles focused at the expected positions). In straight microchannels, the particle focusing depends only on the inertial migration. The shape of microchannel cross-section affects the focusing pattern: particles are focused into a ring in circular pipes [37], focused near the centers of the four channel walls in square channels [9,38], or focused near the centers of the two long channel walls in rectangular channels [39,40,41,42]. Therefore, 3D particle focusing cannot be achieved solely by inertial migration in straight microchannels (Figure 4) [39]. An effective solution is to introduce the drag forces of secondary flows to compete with the inertial lift using special structures [43,44,45,46] or curved channel shapes [47,48,49,50,51,52].

### 3.2. Inertial Particle Separation in Straight Microchannels

**Figure 5:**
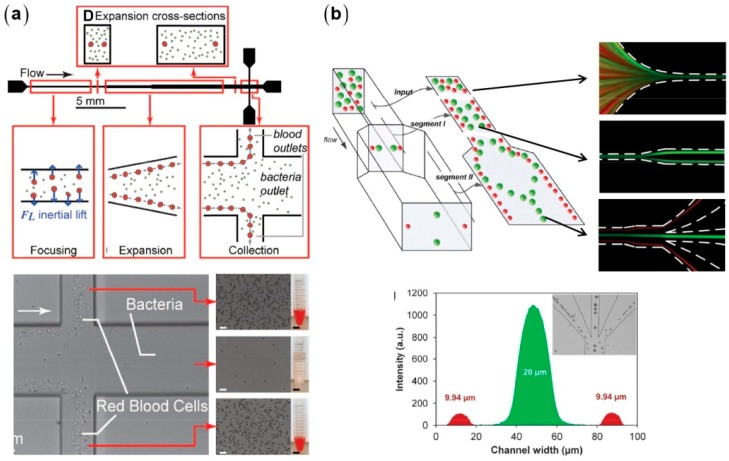
Inertial particle separation in straight microchannels: (**a**) red blood cells and *E. coli* bacteria [53] (Reproduced with permissions from Mach et al., Biotechnology and Bioengineering; published by Wiley Online Library, 2010); and (**b**) polystyrene particles with different diameters [54] (Reproduced with permissions from Zhou et al., Lab on a Chip; published by Royal Society of Chemistry, 2013).

Inertial effects in straight microchannels can be used for particle separation (Figure 5) [53,54], which is based on the size-dependent migration velocities resulting from the different scalings between inertial lift ($a^{4}$) and viscous drag force (a). Rectangular microchannels are more desirable for particle separation due to the less equilibrium positions [38,55]. Mach et al. used a rectangular straight microchannel with a gradually expanded segment to separate *E. coli* bacteria from human blood samples [53]. The blood cells are focused along the side walls and enter into the branched outlets, whereas bacteria remain dispersed and largely flow into the main outlet. The focusing quality of blood cells degrades at large number densities due to the cell-cell interactions, which need to be minimized via the dilution of blood samples, typically requiring that the length of the equivalent single-cell train is less than 50% of the total microchannel length [33].

### 3.3. High-Throughput Particle Transfer and Detection Based on Inertial Microfluidics

**Figure 6:**
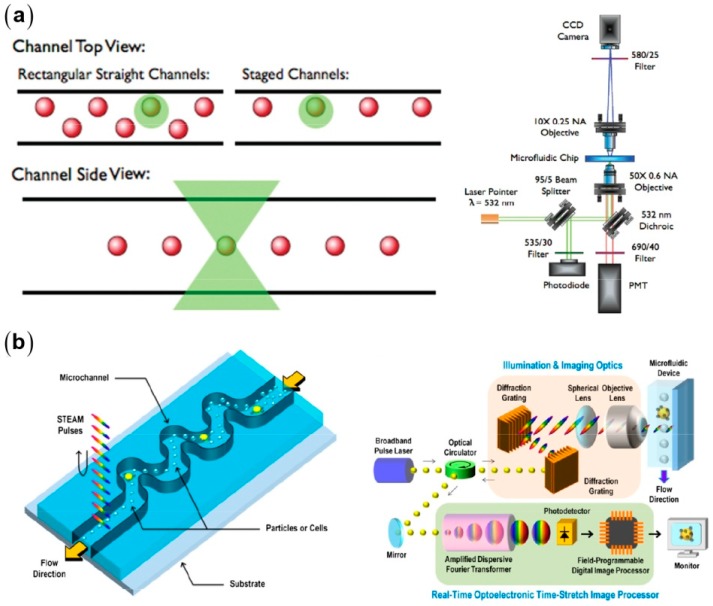
Single-cell imaging flow analyzer based on 3D inertial focusing: (**a**) particle positions relative to the focused laser beam in the cytometer apparatus; and (**b**) a flow analyzer that highlights a microfluidic particle focuser and a real-time imaging system [58,59] (Reproduced with permissions from Goda et al., Proceedings of the National Academy of Sciences of the United States of America; published by National Academy of Sciences of the United States of America, 2012 and Reproduced with permissions from Oakey et al., Analytical Chemistry; published by American Chemical Society, 2010).

The rapid transfer of particles and cells between disparate solutions is important to diverse chemical and biological fields [56,57]. The controllable cell transfer across streams is always challenging as it often requires sophisticated flow control, finely tuned externally applied fields, or precisely manufactured structures. Using a microchannel with shifting aspect ratios (AR=W/H, where W is the channel width and H is the height), Gossett et al. realized simple and controllable cell transfer from the side wall to the centerline [42]. The flow rate ratio of transfer fluid to cell fluid is finely tuned to make the transfer fluid occupy the centerline of the main microchannel. Consequently, cells migrate across the interface and enter the transfer fluid at rates exceeding 1000 particles per second. Inertial microfluidics can be also applied to high-speed CTC analysis via cooperation with other high-speed devices [58,59]. Di Carlo’s group made a portable CTC clinical detection system using a serpentine microchannel as the high speed cell focuser, achieving a throughput of 10^5^ cells per second (Figure 6) [58,59].

### 3.4. Inertial Particle Separation in Curved Microchannels

Dean flow induced in curved microchannel exerts an additional drag force (Dean drag $F_{D}$) on particles, providing a more flexible separation principle. The competition between the inertial lift and the Dean drag results in size-dependent equilibrium positions of particles due to the different force scaling, i.e., $F_{L}\propto a^{4}$ and $F_{D}\propto a$. Dean flows are characterized by Dean number, $De=Re\sqrt{D/R}$, where R is the radius of curvature of the microchannel. The Dean velocity depends on the mainstream flow rate and the curvature of the microchannel $U_{\mathrm{Dean}}\propto De^{2}\eta /\left(\rho D\right)$ [60]. Introducing Dean flow changes the particle migration by two aspects: (1) The Dean flow, parallel to the cross-section, can accelerate the migration toward the equilibrium positions, and thus shorten the microchannel length [48]. (2) The ratio of $F_{L}$ to $F_{D}$ generally determines particle behaviors: (1) $F_{L}>>F_{D}$, particle migration is dominated solely by inertial lift and the focusing pattern is expected to be the same with that in straight microchannels; (2) $F_{L}\ll F_{D}$, particles just follow Dean flows neglecting the inertial lift and therefore no particle focusing can be achieved; and (3) $F_{L}∼F_{D}$, inertial lift and Dean drag synergistically affect the particle migration and lead to different focusing patterns depending on the ratio of $F_{L}$ to $F_{D}$. The applications of the inertial microfluidics lie in the third regime.

**Figure 7:**
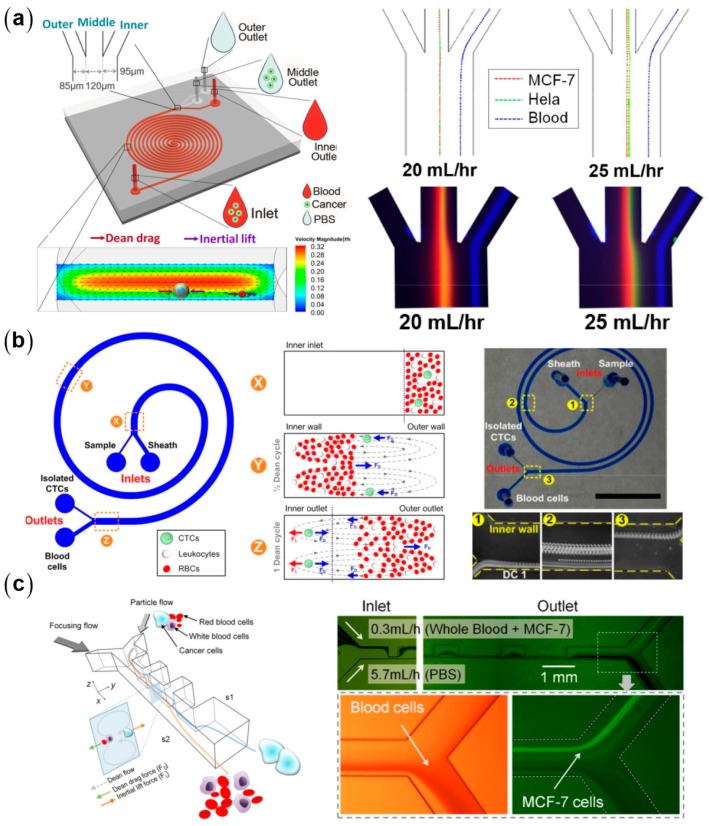
Inertial separation of circulating tumor cells in typical microchannel designs: (**a**) double spiral microchannel [68] (Reproduced with permissions from Sun et al., Lab on a Chip; published by Royal Society of Chemistry, 2012); (**b**) single spiral microchannel [61] (Reproduced with permissions from Warkiani et al., Lab on a Chip; published by Royal Society of Chemistry, 2014); and (**c**) expansion–contraction array microchannel [70] (Reproduced with permissions from Lee et al., Analytical Chemistry; published by American Chemical Society, 2013).

**Figure 8:**
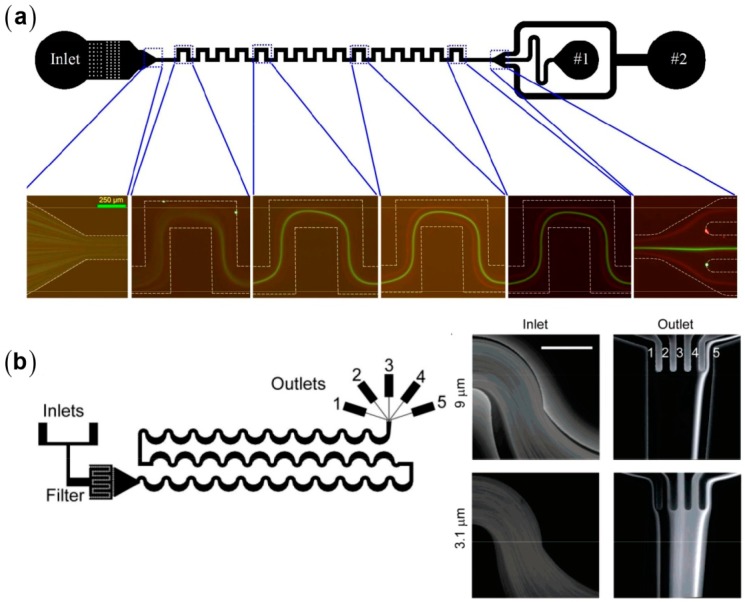
Particle separation in serpentine microchannels: (**a**) the separation of 10 μm and 3 μm polystyrene beads in a symmetry microchannel [65] (Reproduced with permissions from Zhang et al., Scientific Reports; published by Nature Publishing Group, 2014); and (**b**) the separation of 9.3 μm and 3.1 μm polystyrene beads in an asymmetry microchannel [47] (Reproduced with permissions from Di Carlo et al., Analytical Chemistry; published by American Chemical Society, 2008).

There are two types of curved microchannels commonly used for particle separation: spiral (Figure 7) [49,50,51,52,61,62,63,64] and serpentine microchannels (Figure 8) [9,47,65,66]. Using a single spiral microchannel, Kuntaegowdanahalli et al. successfully separated polystyrene (PS) beads with diameters of 10, 15, and 20 μm with an efficiency of 90% [50]. They further separated two types of tumor cells, SH-SY5Y neuroblastoma cells (average diameter of 15 μm) and C6 glioma cells (average diameter of 8 μm), with an efficiency of 80% and a throughput of 10^6^ cells per minute [50]. Using a similar design, Hou et al. separated CTCs from diluted whole human blood with an efficiency of 85% [67]. All the above devices require sheath flows, leading to limited sample throughputs. Sun et al. separated CTCs from human blood with a high throughput of 2.5 × 10^8^ cells per minute and an efficiency of 90% using a high aspect-ratio microchannel with numerical optimization [68,69]. Bhagat and his collaborators designed a spiral microchannel with a trapezoidal cross-section for particle separation with enhanced resolution and throughput [51]. The trapezoidal design redistributes the Dean flow intensities and inertial lift forces, making the focusing patterns more sensitive to the particle size and the flow rate. The equilibrium positions can sharply shift with a large lateral distance at a size-dependent critical flow rate, leading to a large separation distance. This design is further used for high throughput separation of CTCs at a throughput of 56 mL blood per hour and a recovery of 80% [61].

Serpentine microchannels have their curvatures frequently alternated compared with spiral ones, resulting in a more complex competition between the inertial lift and the Dean drag. On the other hand, serpentine microchannels exhibit better parallelizability. Di Carlo et al. adopted asymmetry serpentine microchannel for investigating the inertial focusing behaviors of red blood cells (RBCs) and found that: (1) RBCs suffer no discernable damage during the inertial manipulation; and (2) blockage ratios κ higher than 0.07 are required for successful particle focusing [9]. Based on the asymmetry serpentine design, they successfully separated PS beads with diameters of 9 and 3 μm and isolated platelets from whole human blood [47]. Serpentine microchannels are often designed to be asymmetric to avoid the offset of the counteracting secondary flows in the opposing segments. However, using a symmetry serpentine microchannel, Zhang et al. successfully separated PS beads with diameters of 10 and 3 μm. The 3 μm beads (κ=0.04) were tightly focused in their microchannels with D of 70 μm, which is inconsistent with the previous claim by Di Carlo group [65,66]. This inconsistency indicates an incomplete understanding of inertial focusing mechanisms.

### 3.5. Fundamentals of Inertial Focusing and Recent Development

**Figure 9:**
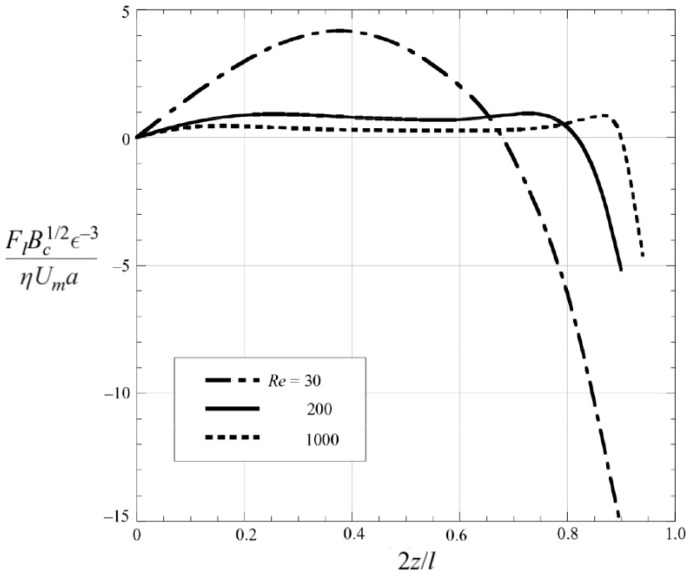
The distributions of inertial lift coefficients at *Re* ranging from 30 to 1000 showing that the lift profile exhibits a concavity change at higher *Re* [78] (Reproduced with permissions from Matas et al., Journal of Fluid Mechanics; published by Cambridge University Press, 2004).

The optimization of inertial microfluidic devices often requires the evaluation of the focusing pattern of targeted particles, which is determined by the lift force distributions. Since Segre and Silberberg observed the inertial particle focusing in 1961 [37], a plenty of theoretical studies have been proposed to reveal its underlying hydrodynamic mechanism [30,31,71,72,73,74,75,76,77]. All these analytical studies were conducted by solving Navier–Stokes equations using the perturbation methods. Investigating the motion of a sphere in a two-dimensional Poiseuille flow, Ho and Leal obtained an explicit formula for the lift force: $F_{L}=C_{L}\rho U^{2}a^{4}$, where the lift coefficient $C_{L}$ is the function of the lateral position and is independent of the detailed undisturbed velocity profile [30]. Their lift formula can successfully explain the inertial focusing patterns in planar or tube Poiseuille flows. However, the restriction of Re≪1 and κ≪1 limits its application to practical situations where Re is finite. Using the matched asymptotic perturbation method, Asmolov extended the applicable *Re* to 3000 (Figure 9) [31]. Asmolov and Matas calculated the lift forces at Re exceeding 1000 with the requisition of $Re_{p}\ll 1$ and found that with the increasing Re, the equilibrium position shifts closer to the channel wall and the magnitude of $C_{L}$ decreases, which is consistent with existing experimental and numerical results [31,78]. However, it is still difficult to directly apply these theoretical studies to the realistic cases of finite-sized particles (intermediate κ), where the particles strongly affect the ambient flow field and cause strong nonlinearity, casting challenge on the theoretical analysis.

**Figure 10:**
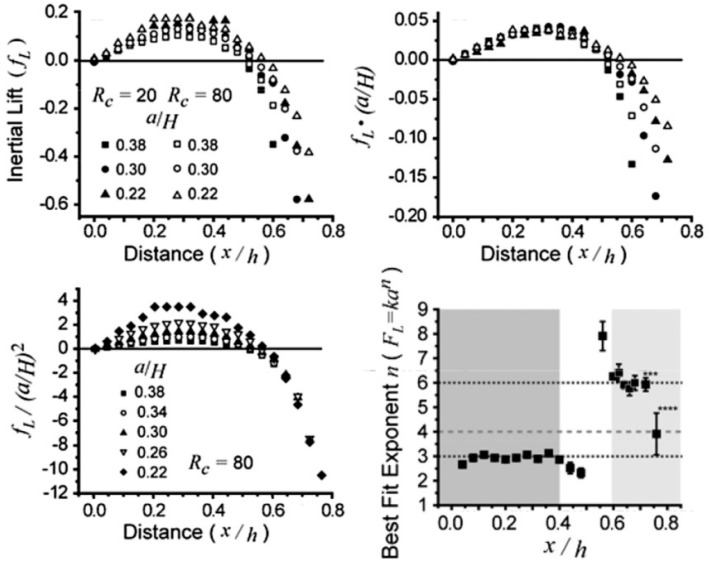
The complex scaling of the inertial lift on finite-sized particles in square microchannels: $F_{L}\propto \rho U^{2}a^{3}/H$, near the channel center and $F_{L}\propto \rho U^{2}a^{6}/H^{3}$ near the channel wall [41] (Reproduced with permissions from Di Carlo et al., Physics Review Letters; published by American Physical Society, 2009).

Direct numerical simulation (DNS) is able to investigate the motions and acting forces of particles without simplified models [79,80]. In DNS, the acting hydrodynamic force on a particle is calculated by integrating the total stress over its surface. Di Carlo et al. obtained a position-dependent scaling for inertial lift in square channels: $F_{L}\propto \rho U^{2}a^{3}/H$ near the channel center and $F_{L}\propto \rho U^{2}a^{6}/H^{3}$ near the channel wall (Figure 10) [41], which is different from the uniform scaling law obtained by theoretical calculations. Using the arbitrary Lagrangian–Eulerian method (ALE), Joseph’s group investigated the inertial lift on a sphere in a slit and a circular tube [81] and found that the lift profile exhibits a convexity change at higher Re (Re>300). Here positive lift force directs toward the channel wall while the negative one directs toward the channel center. At low Re (Re∼O(10)), the lift curve concaves downwards with a positive slope near the channel center and a negative slope near the channel wall. At high Re (Re>300), the convexity becomes more complex: two concave-downwards segments occur at the channel center with the equilibrium positions with a maximum in each segment and a concave-upwards segment lies between the two concave-downwards ones. Asmolov’s theoretical calculation also obtained a similar convexity change for Re>300 [31]. Matas et al. experimentally observed a distinguished double-ring focusing pattern in a tube at Re>760, which can be explained by the convexity change at high Re [78,82].

**Figure 11:**
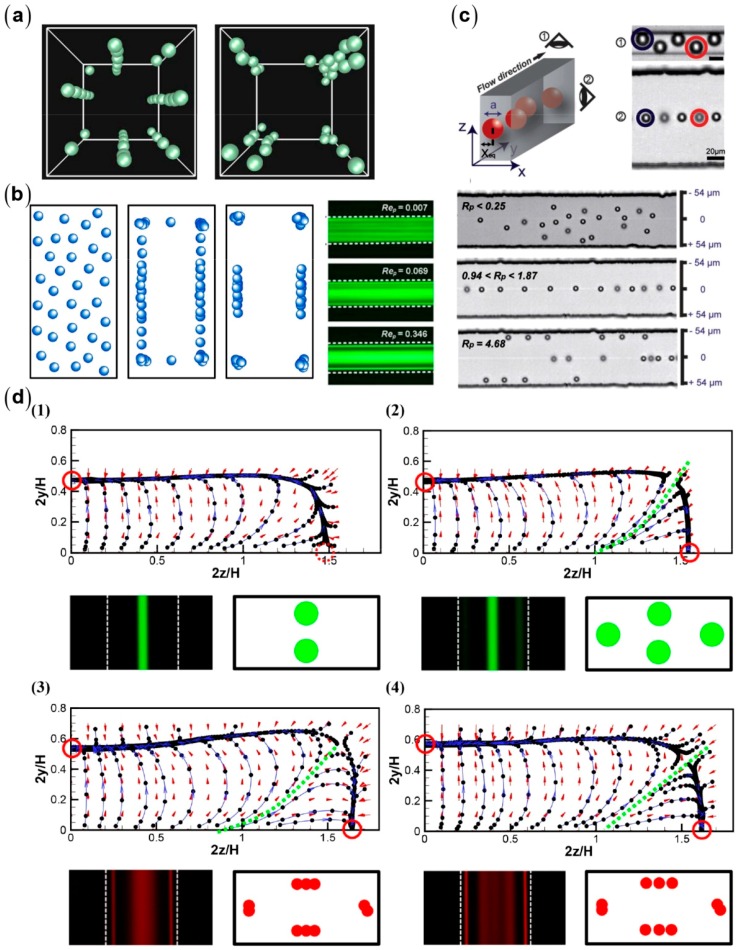
Inertial focusing patterns in rectangular microchannels: (**a**) AR=1, Re=100−500, κ=0.11 [84] (Reproduced with permissions from Chun et al., Physics of Fluids; published by American Institute of Physics Publishing, 2006); (**b**) AR=2, Re=1−100, κ=0.08 [85] (Reproduced with permissions from Bhagat et al., Physics of Fluids; published by American Institute of Physics Publishing, 2008); (**c**) AR=2, Re=0−230, κ=0.2 [39] (Reproduced with permissions from Hur et al., Lab on a Chip; published by Royal Society of Chemistry, 2010); and (**d**) (**1**) Re=100, κ=0.3; (**2**) Re=200, κ=0.3; (**3**) Re=100, κ=0.1; and (**4**) Re=200, κ=0.1 [90] (Reproduced with permissions from Liu et al., Lab on a Chip; published by Royal Society of Chemistry, 2015).

Microchannels fabricated by the planar soft-lithography methods commonly have square or rectangular cross-sections [83]. In addition to the common four off-center focusing positions near each channel wall, more complex focusing patterns have been observed in square microchannels (Figure 11). Using the lattice Boltzmann method, Chun et al. found eight equilibrium positions in square channels at Re=100 [84]. The similar focusing pattern was also observed in the experiments by Bhagat et al. [85]. The four equilibrium positions near the channel centers disappear at Re exceeding 500 [84]. In rectangular microchannels, particles are typically focused near the centers of the long walls [39,40,41,42,53,86,87]. This reduction in equilibrium position makes rectangular microchannels more favorable for particle focusing and separation. However, six or even eight positions have also been observed in rectangular microchannels with similar AR [85,88]. Zhou et al. experimentally showed that the rotation-induced forces play a role in particle migration toward the centers of the long walls [89]. However, the rotation-induced force always directs toward the center of the channel walls, and thus cannot explain the multiple equilibrium positions near the long walls. Gossett et al. numerically investigated the inertial migration of particles at *Re* = 80 in a microchannel with AR=2 [42]. They found two stable equilibrium positions centered at the long walls and two unstable ones centered at the short walls, which is consistent with the typical focusing pattern.

**Figure 12:**
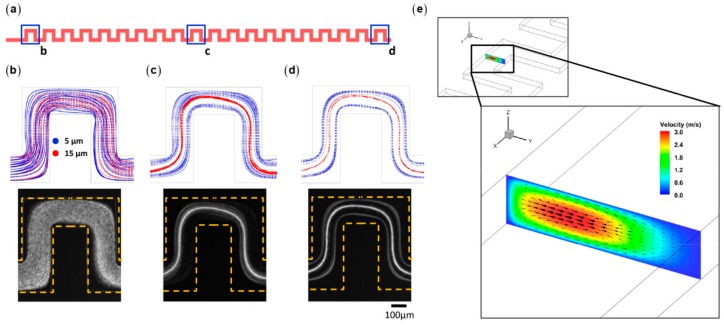
The particle trajectories in a serpentine microchannel calculated by Lagrangian particle tracking based on the explicit lift formula: (**a**) the schematics of the serpentine microchannel; (**b**–**d**) simulation (top row) and experimental observation (bottom row) of particle trajectories are shown at the: (**b**) 1st unit; (**c**) 10th unit; and (**d**) 20th unit; and (**e**) the Dean vortex forming in the zigzag section at the flow rate of 50 mL/h (*Re* ≈ 120) [91] (Reproduced with permissions from Liu et al., Lab on a Chip; published by Royal Society of Chemistry, 2016).

Using combination of numerical simulation and experiments, Hu’s group systematically investigated the inertial focusing patterns for a wide range of *Re*, κ, and *AR* [90]. The typical focusing near the centers of the long walls in rectangular microchannels is obtained at relative low *Re*. New stable equilibrium positions will emerge at high *Re* due to the stabilization of the sub-stable equilibrium near the centers of the short walls or due to the attractive lift forces near the long walls. The critical *Re* decreases with κ for fixed *AR* and decreases with *AR* for fixed κ. Although it has provided insights for the fundamentals of inertial focusing, DNS is still burdensome when applied to practical long microchannels with complex geometries. Hu’s group proposed a fitting formula for the inertial lift on a sphere drawn from DNS data obtained in straight channels [91]. The fitting formula is a function of the parameters of the local flow field, and thus is adaptable to complex microchannels. Being implemented in the Lagrangian particle tracking method, the formula is used to fast predict particle trajectories in some widely used microchannel types (Figure 12).

## 4. Viscoelastic Manipulation of Particles

High pressure is needed to generate an inertial flow in a scaled-down microchannel for the manipulation of smaller particles [92]. By contrast, deterministic particle migration can be obtained in viscoelastic fluids even at very low flow speeds, avoiding high pressure drops across the microchannels [93,94]. In addition, the synergetic combination of inertial lift and elastic lift can achieve a real 3D focusing at the microchannel centerline [95]. Most naturally-occurring biochemical samples, such as blood, lymph, saliva, and protein solutions, are viscoelastic [96]. Therefore, the elasto-inertial microfluidic particle manipulation may have wide applications in many biochemical fields.

### 4.1. Fundamentals of Particle Migration in Viscoelstic Fluids

Viscoelastic microfluidics typically use the aqueous solutions of synthetic or naturally-occurring polymers as carrier mediums. The most commonly used polymers are poly(ethylene oxide) (PEO) and poly(vinylpyrrolidone) (PVP), which have good water solubility. In a sheared or stretched flow, the polymer chains are elongated along the flow direction, causing stress anisotropy, i.e., the non-zero normal stress differences [97]. The first and second normal stress differences are defined as $N_{1}=\sigma_{xx}-\sigma_{yy}$ and $N_{2}=\sigma_{yy}-\sigma_{zz}$, respectively (here *x*, *y*, and *z* denote the direction of the flow, velocity gradient, and vorticity, respectively). The non-dimensional Weissenberg number (*Wi*) characterizes the fluid elasticity, which is the ratio of the first normal stress difference to the viscous shear stress. *Wi* can be expressed as $\dot{\gamma }\lambda$ using Oldroyd-B constitutive model, where $\dot{\gamma }$ is the shear rate and λ the relaxation time. Using the regular perturbation method, Ho and Leal calculated the lift force on a small sphere (κ≪1) suspended in an inertialess Poiseuille flow of a second-order fluid, showing that the elastic lift stems from the imbalance of normal stress difference over the sphere size [93], i.e., $F_{e}∼a^{3}\nabla N_{1}$ (here the effect of $N_{2}$ is neglected as $\left|N_{2}/N_{1}\right|\leq 0.1$ for most polymer solutions [98]). Oldroyd-B model gives the positive value of $N_{1}=2\eta \lambda {\dot{\gamma }}^{2}$, indicating compressive normal stresses on the sphere surface that become stronger with increasing local shear rate. In a non-uniformly sheared flow, a net lift force will drive particles toward the positions where $\dot{\gamma }$ have minimums.

### 4.2. Particle Manipulation in Viscoelastic Microfluidics Devices

**Figure 13:**
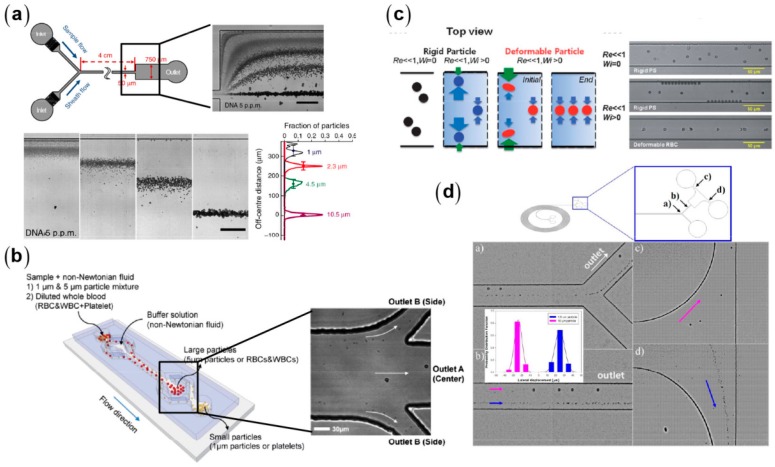
Viscoelastic particle separation: (**a**) separation of PS particles of different diameters with the aid of sheath flow [105] (Reproduced with permissions from Kang et al., Nature Communications; published by Nature Publishing Group, 2013); (**b**) separation of red blood cells and platelets with the aid of sheath flow [101] (Reproduced with permissions from Nam et al., Lab on a Chip; published by Royal Society of Chemistry, 2012); (**c**) separation of rigid and deformable cells [99] (Reproduced with permissions from Yang et al., Soft Matter; published by Royal Society of Chemistry, 2012); and (**d**) separation of PS particles with different diameters in curved microchannel [103] (Reproduced with permissions from Lee et al., Scientific Reports; published by Nature Publishing Group, 2013).

In square or rectangular microchannels, particles are focused at the center and four corners [95,99,100]. For *Re* of O(1), particles are only focused along the channel centerline due to synergetic combination of the elastic lift and the wall-induced lift [95,101,102]. This simple focusing pattern can realize particle separation by size difference via sheath flows, curved microchannels, or embedded structures (Figure 13). Using a sheath flow to prefocus different-sized particles along the sidewall, they can be separated due to lateral velocity difference determined by the balance between the elastic lift (*F_e_* ~ *a*^3^) and viscous drag (*F_d_* ~ *a*). Separation using sheath flow can work at a relatively wide range of flow rates, but has limited throughputs due to high ratio of sheath flow to sample flow rates. Therefore, whether or not using sheath flows depends on the specific separation task. In addition, inducing secondary flow in curved microchannels to compete with elastic lift can also achieve size-based separation of particles [103,104].

**Figure 14:**
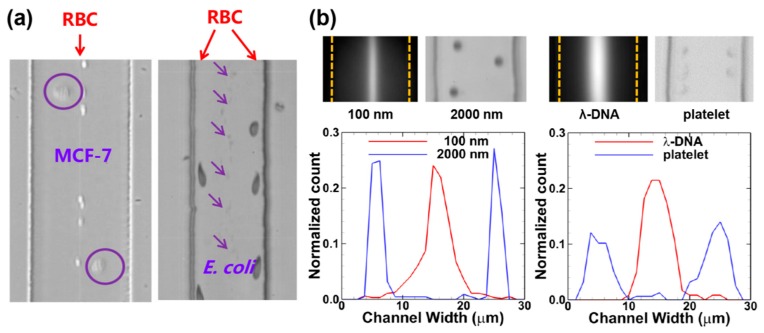
Sheathless separations of diverse binary bioparticle mixtures: (**a**) the separation of the mixture of MCF-7/RBC (left) and RBC/*E. coli* (right) [106] (Reproduced with permissions from Liu et al., Analytical Chemistry; published by American Chemical Society, 2015); and (**b**) the separations of smaller binary mixtures of 100 nm/2000 nm polystyrene spheres (left) and λ-DNA/platelet (right) [91] (Reproduced with permissions from Liu et al., Analytical Chemistry; published by American Chemical Society, 2016).

Most viscoelastic particle separations are based on the size-dependent migration velocities. The particle size itself can also affect the focusing pattern. Liu et al. found that 15 μm particles are focused at the both sides of the centerline of a 50-μm-high microchannel at optimized flow rates [106]. By contrast, 5 μm particles are always focused closer to the centerline than 15 μm ones at all investigated flow rates. The mechanism of the off-center focusing relates to the strong coupling between the large particles and the ambient flow field. When a large particle deviates from the channel centerline, the major portion of the viscoelastic fluid chooses to flow through the larger gap between the particle and the channel wall [107]. Therefore, the shear rates and the resultant compressive normal stresses are intensified at the near-center side of the particles, and consequently the particles are driven toward the wall [80]. This off-center focusing pattern occurs for a wide range of *AR* and is determined by the ratio of particle diameter to the narrowest channel dimension. In addition, this off-center focusing can be obtained at a wide range of scales, from macro- to nanoscales, realizing sheathless separations of diverse particles including microparticles, CTCs, RBCs, bacteria, nanoparticles, and biomoleculars (Figure 14) [91,106].

Viscoelastic focusing generally works at 1–2 orders of magnitude slower flow rate than inertial focusing due to the focusing degradation at higher flow rates [95]. There are two defocusing mechanisms and each of them corresponds to a solution. One is that the arising shear-gradient-induced lift drives particles away from the channel centerline, indicating that elasticity needs to be enhanced to balance the shear-gradient-induced lift. Kang et al. used a highly elastic medium (5 ppm λ-DNA solution, λ = 0.14 s) to achieve tight particle focusing over a wide range of flow rates (0.005–2 mL/h) [105]. The other mechanism is that the flow is destabilized when elasticity solely dominates at high *Wi* [108,109]. If fluid inertia and elasticity simultaneously dominate, the flow will keep stable even at the regime in which turbulent flow in the Newtonian fluid is observed. Lim et al. reported particle focusing at extremely high flow rates (1200 mL/h, *Re* = 4630, *Wi* = 566) using a medium with low relaxation time (0.1 *w*/*v* % hyaluronic acid (HA) solution, λ = 8.7 × 10^−4^ s) [102]. There seems to be contradiction between these two mechanisms, implying an inconclusive understanding of particle migration in viscoelastic medium.

Considering the high price of DNA and HA, existing studies are more focused on optimizing the performance of cheap synthetic polymers, such as PEO and PVP. Liu et al. found that an optimized polymer solution for particle manipulation should have low viscosity, minimized shear shinning, and strong elasticity. The shear thinning is ubiquitous for polymer solutions, especially at high polymer concentration or large molecular weight [106]. Therefore, a trade-off should be properly made between the minimized shear thinning and the necessarily strong elasticity. Liu et al. used naturally denaturized PEO solution (4 × 10^6^ Da) to successfully focus 5 μm particles in a 50 μm high microchannel at throughputs one order of magnitude higher than those of newly prepared PEO solutions [106] (Table 2). Compared with its newly prepared counterpart, the denaturized PEO solution has lower viscosities and much weaker shear thinning and still remains highly elastic, eliminating the focusing degradation at higher flow rates.

## 5. Conclusions

High-throughput particle manipulation is of significance in diverse applications in biological, biomedical, and environmental fields, requiring the ability to handle large volumes of samples. The last decade has seen the rise of inertial microfluidics as a novel tool for high-throughput and label-free particle manipulation utilizing the controllable particle migration driven by inertial lift. However, the pure inertial manipulation achieves its optimal performance often at a narrow flow rate range and faces challenges in handling nanoparticles in down-scaled channels, limiting its widespread usage for the diverse applications. Introducing the viscoelastic effects of carrier medium, the elasto-inertial microfluidic devices can intensively extend the working flow rate range and reduce the applicable particle size. However, there is still a lack of conclusive understanding of particle motion in viscoelastic medium, leading to poor guidelines and difficulties in the design of elasto-inertial microfluidic systems. Systematic studies are urgently needed to elucidate the effects of complex rheological properties, channel geometry, and particle properties on the focusing pattern. Despite the success in laboratories, there are not many reported handling of clinical samples based on the inertial/elasto-inertial concept. To achieve better adaptability to realistic applications, a promising strategy is to couple inertial/elasto-inertial effects with other externally applied physical fields, such as electric, magnetic, and acoustic ones, to further improve the purity and resolution.

## Figures and Tables

**Table 1:** Typical force field types used for microfluidic cell manipulation.

Technique	Separation Marker	Mechanism	Force Scaling with Diameter
Acoustophoresis	a, ρ, α	Ultrasonic sound wave	$a^{3}$
Dielectrophoresis	a, ε, σ	Non-uniform electric field	$a^{3}$
Magnetophoresis	a, χ	Magnetic field	$a^{3}$
Optophoresis	a, n	Optical field	Optical gradient force: $a^{3}$ Scattering force: $a^{6}$
Centrifugation	a, ρ	Centrifugal force	$a^{3}$

Note: a: diameter; ρ: density; α: compressibility; ε: permittivity; σ: electric conductivities; χ: magnetic permeability; n: reflective index.

**Table 2:** Comparison of recent works on particle focusing and separation using viscoelastic solutions.

Study		Minimum Particle Size for Successful Manipulation (μm)	Minimum Effective Blockage Ratio	Sample Flow Rate (μL/h)	Focusing Efficiency (%)	Separation Efficiency (%)	Channel Geometry and Footprint	Journal	Manipulation Type
**Microparticle**	Leshansky et al. [94]	5	0.11	400–2000	>95	N/A	Straight; N/A	Physical Review Letters	Viscoelastic focusing
	Kang et al. [105]	5.8	0.116	5–2000	~100	N/A	Straight; 50 mm long	Nature Communications	Viscoelastic focusing/separation
	Lim et al. [110]	6	0.075	3 × 10^6^	~90	N/A	Straight; 35 mm long	Nature Communications	Elasto-inertial focusing
	Lu et al. [111]	3	0.06	~O(100)	N/A	~100	Straight; 20 mm long	Analytical Chemistry	Elasto-inertial pinched flow fractionation separation
	Liu et al. [106]	1	0.063	10–3000	~100	~100	Straight; 30 mm long	Analytical Chemistry	Elasto-inertial focusing/separation
	Lee et al. [103]	1.5	0.038	~O(100)	N/A	~100	Spiral; 500 mm long	Scientific Reports	Viscoelastic separation
	Yuan et al. [112]	3	0.053	600–4800	~100	~100	Straight; 48 mm long	Lab on a Chip	Elasto-inertial focusing/separation
	Nam et al. [101]	5	0.1	30	N/A	~100	Straight; 25 mm long	Lab on a Chip	Elasto-inertial focusing/separation
	Yang et al. [95]	5.9	0.118	40–320	N/A	>95	Straight; 40 mm long	Lab on a Chip	Elasto-inertial focusing
**Nanoparticle**	De Santo et al. [113]	0.2	0.04	0.002–0.016	85	N/A	Straight; 100 mm long	Physical Review Applied	Viscoelastic focusing
	Kim et al. [114]	0.2	0.04	<0.96	Low: multiple streams	N/A	Straight; 40 mm long	Lab on a Chip	Viscoelastic focusing
	Liu et al. [91]	0.1	0.014	0.32–2.45	84	>95	Double spiral; >60 mm long; 3 × 3 mm^2^	Analytical Chemistry	Viscoelastic focusing/separation
